# A Training Program to Enhance Disaster Preparedness of Group Companies in the Tokyo Metropolitan Area

**DOI:** 10.3390/ijerph16234871

**Published:** 2019-12-03

**Authors:** Noriko Sudo, Gengaku Mashiro, Shigeru Beppu, Risa Hakamata

**Affiliations:** 1 Natural Science Division, Faculty of Core Research, Ochanomizu University, Tokyo 112-8610, Japan; 2 BC Research Institute, Tokyu Facility Service Co. Ltd., Tokyo 158-8539, Japan; gengaku.mashiro@tokyu-facility-service.co.jp (G.M.); risa.hakamata@tokyu-facility-service.co.jp (R.H.); 3 Food Processing Technology, Graduate School of Niigata University, Niigata City 950-2181, Japan; beep@bl.mmtr.or.jp

**Keywords:** disaster preparedness, stockpile, disaster training

## Abstract

Many business continuity (BC) plans do not mention food and water for BC personnel. Moreover, the BC relies on the assumption that, during an emergency or crisis, employees’ basic needs and personal hygiene are satisfied. Although no one can engage in BC without these supplies, literature regarding companies’ disaster stocks for their employees is limited. We evaluated the current situation of companies’ stockpiles of food and other supplies and what their employees thought about them after participating in a newly-developed overnight training program that allows the participants to experience situations that they would encounter in a disaster. Thirty-three employees from eight companies in Tokyo participated in the program. Seventy-five percent of the participants’ companies had food stocks for three days as instructed by the Tokyo Metropolitan Government but, after eating four stock meals, 81.3% of the participants thought it would be better if this provision were improved. The stock rate for bedding was 62.5% but less than 30% of companies stocked both blankets and mats, as suggested by the Sphere Standards. There were several people who complained of sleeplessness and a poor physical condition the next morning and this could be an obstacle in the BC.

## 1. Introduction

Companies must continue to do business, even in disasters. Discontinuation of business could result in loss of earnings and credit and affect corporate activities after the disaster. Studies have shown that publicly traded companies without a business continuity plan (BCP) or with an inadequate plan reduce their market viability [1]. Business continuity (BC) is important not only for company profits but also in terms of corporate social responsibility. It is the BC of each company that supports rescue and assistance by local and central governments through providing products and services. Companies must ensure BC in order to provide relief supplies and essential services such as transportation, mass communication, security, construction, retail, and facility management to support the lives of those affected by the disaster [2].

Under the 2016 National Resiliency Action Plan, the Japanese government set the goal of ensuring that 100% of all large enterprises and 50% of all medium-sized enterprises have a BCP by 2020. BCP decreases or eliminates the disruption to employees and profitability. Although staff are included as a BCP resource, many BCPs do not mention food and water for the BC personnel [3]. The BC relies on the assumption that, during an emergency or crisis, employees’ basic needs such as food, water, and personal hygiene are satisfied [4]. Lack of attention to the needs of health care has been identified as an obstacle to effective crisis management in an earthquake [5].

Affected residents in evacuation shelters that are operated by villages, towns, or cities can receive assistance by municipal or prefectural governments, but private sectors are required to carry out self-reliance efforts, even in disasters. Therefore, they must stock necessary items on their own. Critical facilities, such as hospitals, are recognized as playing a crucial role in the recovery of the population after a disaster and their stocks of drinking water and food have been well investigated [6,7,8]. On the other hand, literatures on companies’ disaster stocks for their employees is limited [9]. Therefore, the first purpose of this study is to evaluate the current situation of companies’ stockpiles of food and other supplies.

In addition to the personnel who are engaged in a BC, any other employees could also get stuck at the workplace during a disaster. According to the World City Risk 2025 by the Centre for Risk Studies, Tokyo has the highest risk of flooding and tsunamis and the sixth highest risk of earthquakes among major cities in the world [10]. The Japanese government warned that the probability of an earthquake directly hitting the Tokyo metropolitan area within the next 30 years was as high as 70% [11]. It is estimated that the magnitude will be 7.3 and will result in about 3 million evacuees [12]. One of the big problems specifically caused by an earthquake in big cities such as Tokyo is the mass generation of stranded persons. The Tokyo Metropolitan Government asks citizens to refrain from traveling immediately after a disaster since crowds attempting to walk home due to suspension of public transportation could inhibit emergency vehicles such as ambulances from reaching their destinations due to the streets being flooded with people.

In order to prevent employees from heading home all at once, companies need to allow them to stay at their workplaces until the situation settles. The Tokyo Metropolitan Government Ordinance on Measures for Stranded Persons [13], which was established after the Great East Japan Earthquake in 2011, mandates organizations in Tokyo to stockpile three days of supplies including drinking water and food for all members. A questionnaire survey by the Tokyo Chamber of Commerce and Industry, however, showed that only half of the companies had the stipulated 3-day stockpile [14]. One reason for this might be that they do not understand the importance of storing food and other supplies in the workplace, despite it not being possible to continue any activity in a disaster without food and water, toilets, and accommodation. It is reported that while the majority of businesses took only a ‘few’ or ‘some’ preparedness measures before Hurricane Rita, those business that experienced impacts and losses due to this event are now taking greater preparedness measures [15]. In order to remind them of the importance of preparedness, overnight training sessions are conducted by schools [16] and municipalities [17] to give people the experience of living through a disaster using limited stockpile items.

Among many settings, stockpiles by companies are especially important since they are not only for affected employees but also for workers helping other people through the BC. They contribute to saving victims and reconstructing society. Therefore, we developed a training program for company employees aimed at letting them experience situations that they will encounter in a disaster so that they can think about necessary preparations.

It is said that promoting preparedness for earthquakes can be difficult, especially given the infrequent and varying nature of major events [18]. Disaster experience has a significant and positive impact on business disaster preparedness, and the degree of lifeline loss can be a reasonable indicator of the disaster experience of business organizations [19]. Since the number of people who have directly experienced a large earthquake is limited, our training program provides simulated experience of being affected by an earthquake by banning the use of tap water, electricity, and flushing toilets.

Emergency exercises are considered an important and integral part of emergency preparedness activities. However, little is known about whether these exercises are effective at improving individual and/or organizational preparedness for responding to emergencies [20]. The second purposes of this study are to determine what employees thought about their companies’ stockpiles after participating in the simulated training program and to identify gaps between this provision and the ideal situation. We also present lessons learned from the training experience, as reflected in participants’ comments. Informed by this feedback, we offer recommendations aimed at improving stockpiles.

## 2. Materials and Methods

### 2.1. The Training Plan

Resources and staff experience are things to consider in emergency response and BC [1]. Companies need to consider what resources are available and what will be required and if anyone on staff has experience. We developed a training program focusing on these two elements: Resources and experience. Before the training, we administered a preliminary questionnaire to ask about the company’s stockpiles in order to let the participants know what resources are available beforehand. We required the participants to stay overnight since spending one night and two days only with the stocked items could make them noticed what resources will be required in disasters. We also limited the availability of lifelines in order to mimic the disaster situation for experiential learning. The participants were asked to write their experiences, observations, and reflections on the reflection sheet since written reflections can help them to think critically, increase their active involvement in learning, understand complex situation, and enable them to learn such experiences [21].

### 2.2. Contents of the Training Program

Table 1 shows the contents of the two-day training program. The seats designated to participants formed islands of four to five people in a group, and three liters of water in PET bottles (one 2 L bottle and two 500 mL bottles) were placed on each group’s table, with each person’s name being pasted on the bottle. Since the water supply could be cut off in a disaster, only this amount of water was allowed for drinking, cooking, and brushing teeth in a day, and new purchases were disallowed. However, drinks carried by individuals could be used, just like in an actual disaster.

At the orientation, the first author explained the division of work among groups (Table 2). After selecting the work they wanted to do, each group was left to take out the stockpiled goods (Table A1) from the storeroom of the training room.

Since the power could be cut off in the event of a disaster, only lanterns could be used from this time onwards, except for during lectures when the ceiling lights were turned on. The arrangement of the lanterns in the classroom and toilet was left to the discretion of the lighting team (Table 2). Four meals, from lunch on Day 1 to lunch on Day 2, were brought along by the participants from their own companies’ stockpiles (three meals for those who did not stay overnight). The use of cassette stoves prepared by the secretariat was only allowed if heat sources had been stockpiled at their workplaces. If water was required to cook the food, e.g., hot water for boiling food in retort pouches or for returning pregelatinized rice to its original form, the use of water from the individual’s own PET bottle was allowed. The meal team was responsible for installing the cassette cylinders in the stoves and setting up the pot and kettle for boiling water. From 11:15 a.m. to 6:30 p.m. on Day 1, the water supply to the toilet and washbasin was also stopped. The ceiling lights on the whole floor where the training room was located, including those in the corridor/bathroom, could not be used from 5:30 p.m. on Day 1 until the next morning.

In the event of a disaster, public transportation will stop running, so it will be difficult to return home. Therefore, the participants stayed overnight in the training room. The accommodation team was responsible for distributing bedding, such as air mats and sleeping bag sheets, as well as arranging the sleeping layout.

### 2.3. Participants

We conducted the training program as the third among the five BC training program sessions. In all, 33 executives from eight companies, including Tokyu Facility Service, participated in this program.

The main office of Tokyu Facility Service is in the Tokyo metropolitan area and its main business is facility management service mainly in surrounding areas. They also manage facilities that have the role to address the needs of stranded persons when a disaster occurs. The number of this stranded persons is expected to be so large. For this reason, the employees who work there need to engage in activities to help people of various ethnicities, genders, and age groups. This effort will lead not only to the BC of the company but also to the contribution for the community. Therefore, we called for participation to Tokyu Facility Service and its associated companies.

Also, this training targeted executives. Because we thought that if the executives understood the importance and necessity of BC through this training, we could procure support and funding that are essential for the activities of BC and thus aid in conducting the activities smoothly.

Tokyu Facility Service established the BC Research Institute in 2018. The aim is to improve their survival capabilities. And through this, they want to strengthen employees, companies, and community and to continue functioning the community even through in the disaster.

The BC Research Institute called for participation for the training by sending emails and visiting companies. Considering the capacity of a training room, we limited the number of participants was 40. The businesses of the eight companies participated in the training were: Railway business, hotel and resort service, station retail services, supermarkets, mall developers, construction, security services, and mass communication.

### 2.4. Data Collection

#### 2.4.1. Preliminary Questionnaire

The BC Research Institute sent the preliminary questionnaire to the participants by email and asked them to return the completed questionnaire before the start of training. Its purpose was to let the participants know the stockpiling status of their own company. The questionnaire was designed to enable us to compare the results to the previous studies by using similar questions [22]. Items included in the questionnaire are shown in Appendix A.

#### 2.4.2. Reflection Sheet

We collected a reflection sheet during the training and 32 participants submitted it (response rate = 97.0%). Its purposes were to investigate (1) what the participants thought about their current stockpiles, (2) whether the individual wanted to change the stockpile, and (3) whether the individual understood the importance of selecting the stockpile items while taking their actual use into consideration instead of just the purchasing and management circumstances.

### 2.5. Statistical Analyses

The number of valid responses varied by question. The associations between participation in overnight training and categorical variables were examined by two-by-two frequency tables. Since expected frequency in two cells was below five, associations between the two binary variables were examined by Fisher’s exact test. The Mann–Whitney U test was used when the amount of water remaining was compared by whether the participants took part in the overnight training. The Mann–Whitney U test is a non-parametric test for continuous variables that are not normally distributed. It compares medians between two groups. The level of statistical significance was set at 5%. SPSS Version 24 (Japan IBM, Tokyo, Japan) was used for all statistical analyses.

### 2.6. Ethical Considerations

The study protocol was reviewed and approved by the institutional review board of Ochanomizu University (Notification No. 2018-84). Written informed consent was obtained from all participants.

## 3. Results

### 3.1. Preliminary Questionnaire

Thirty-three participants took part in the training, and all of them sent back the preliminary questionnaire (response rate = 100%).

#### 3.1.1. Participation in the Overnight Training

Fourteen people (42.4%), including all the three women, answered that they were not going to stay overnight. The reasons included “worried about my physical condition,” “do not feel like it,” “work circumstances,” “family matters,” and “personal reasons.”

#### 3.1.2. Stockpiles of Foods and Toilets

Table 3 shows the current food stock situation of at the participants’ companies. Two participants who chose “Others” said “There are food stockpiles, but I do not know what items are stocked unless I ask.” Only one respondent’s company did not have a food stock. The amount of food stockpiled for each person was nine meals for the first quartile, median and mode values, while the third quartile and the maximum value were both 21 meals.

Regarding the per capita amount of drinking water, the range was 1.5 to 10 L, and the median (interquartile range) was 9.0 (2.0, 9.0) L. Twenty-six persons (78.8%) responded that cassette stoves and other heat sources for cooking were stockpiled.

Table 4 shows the combination of stocked foods that were brought along with the participants. Since they were from the group companies, the same products were often observed.

Thirty people (90.9%) answered that their companies stocked disposable toilets for disasters.

### 3.2. Reflection Sheet

#### 3.2.1. What the Participants Think about Their Current Food Stocks

After finishing four meals with their stocked foods only, 26 participants (81.3%) answered “It is better to improve food stocks in the future.” Table 5 shows the answers given to the question of how food stockpiles should be changed. Comments such as “It is better to stock powdered coffee and tea bags” were given in the open-ended response column. There were also comments on luxury drinks like “It would be even better to add luxury food and drink like sweets and coffee,” “I get tired of beverages that consist of water only,” and so on. When we also asked them about the barriers to improvement, the most selected multiple answer was “storage space” (88.5%), followed by “budget” (69.2%), and then “inventory management” (42.3%).

Table 6 shows the results of asking all the participants what is important when selecting foods to stockpile.

#### 3.2.2. Living without Using Running Water and Electricity

At the time that the reflection sheet was submitted, the amount of water remaining was as follows: Minimum value = 1000 mL; maximum value = 2950 mL; mean ± standard deviation = 2274 ± 472 mL. When the results were compared based on whether the participants took part in the overnight training or not, there was no significant difference, but the amount remaining was lower for those who participated (median: 2275 vs. 2500 mL). Table 7 shows the results of asking the participants to freely express their thoughts and observations of living without the use of running water or lights. Twenty-seven participants gave one to three comments. Similar responses were grouped together.

#### 3.2.3. Stockpile of Bedding

Table 8 presents the status on the stockpiling of bedding at the participants’ companies. There were ten participants (31.3%) who did not know whether their company had a stockpile or not. Twenty-two participants (73.3%) responded that “Bedding should be stockpiled or stocked items should be changed in the future.” Upon being asked what they should stockpile, ten people chose “cardboard bed,” which is currently being stockpiled at only one company, while seven people chose “pillow,” which was not stocked at all.

While 83.3% of participants who stayed overnight said “mats” should be stocked, only 33.3% of those who did not stay had this answer (*p* = 0.032). In contrast, “cardboard bed” was significantly less selected by those who participated in the overnight training (25.0% vs. 77.8%, *p* = 0.030).

When they were asked about the “barriers to stockpiling bedding or changes in the stocked items,” the response chosen the most was “storage space” (90.9%) followed by “budget (cost)” (68.2%) and “inventory management” (18.2%). Table 9 revealed that participants in the overnight training came to realize the importance of mats.

#### 3.2.4. Numeric Evaluation of the Current Preparation of Meals, Toilets, and Accommodation

When the participants were asked about their thoughts on the current level of preparation for meals, toilets, and accommodation on a scale of 1 “insufficient” to 10 “sufficient,” the median value (25th percentile, 75th percentile) for meals, toilets, and accommodation was found to be 7.60 (6.25, 8.55), 7.05 (5.00, 8.20), and 5.70 (3.43, 7.55), respectively.

#### 3.2.5. Thoughts and Observations after Having Carried out the Work Assigned to the Teams

Table 10 shows the results of asking the participants to freely express their thoughts and observations after having carried out the work assigned to the teams.

## 4. Discussion

### 4.1. Awareness of Stockpiling Status of their Companies

About 30% of the participants did not know whether any bedding was stockpiled or not at their business enterprise. Regarding the food stocks, almost all participants knew about this beforehand as they had been asked to bring them along. However, it is possible that they might not have known about the stocks if not for this task. Pre-assessment of preparedness is an important component of a training designed to increase disaster preparedness [23]. In a postal survey to municipalities, which are responsible for direct support to their affected residents, 7.3% of respondents did not know the items and quantities of stockpiled foods and water indicated in the regional disaster prevention plan [24]. The first step of a disaster response is to be aware of the amount of goods that one’s own organization has stockpiled [1]. Since the number of people who can go to work in a disaster is limited, it is necessary to create a system that everyone can carry out without relying on a person in charge.

### 4.2. Food Stocks

As for quantity, 75% of the companies stocked three meals per day for three days. Three-day supply of nonperishable food for individual preparedness is often recommended [25]. The newest guide for stockpiles issued by the Japanese Ministry of Agriculture, Forestry and Fisheries in 2019, however, recommends households to stock foods “at least for three days, if possible, for one week” responding to frequent natural disasters in recent years all over the country [26]. It is difficult for business establishments to increase the amount of food stocks since they cannot apply a rolling stock method recommended by the government. Unlike in households, in companies, the buyer (disaster prevention division) and consumer (employees) of stocked foods are different and it is difficult to consume expired stocked foods in daily dietary life.

**Figure 1:**
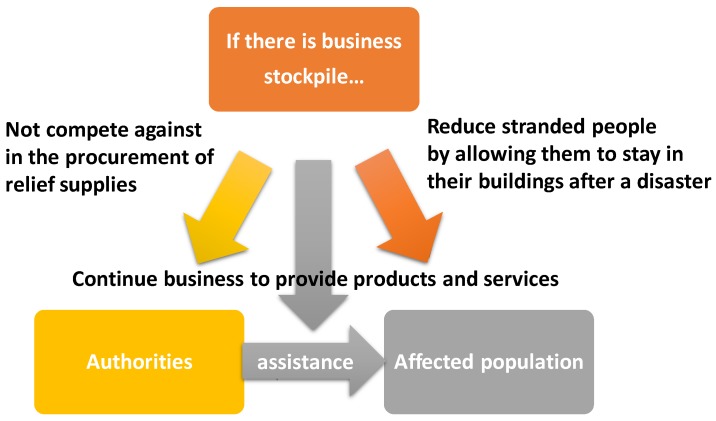
Conceptual diagram how business stockpiles affect general disaster response.

Crises and disasters are low probability events, but they place large demands on resources [27]. In order to avoid competition against increased demands on relief supplies, collaboration between businesses and public sectors are required [28]. Private sectors should not disrupt mass care by the local governments. They should stock basic commodities at normal times because trying to procure a large number of products could disrupt mass care by authorities. Stocks in businesses could also contribute to reduction in floods of people on the streets by allowing them to stay in their buildings after a disaster, making relief activities easy (Figure 1).

As for quality, in a previous study for companies in the Tokyo metropolitan area, the percentages that stocked accompanying dishes such as “main dishes” and “side dishes” were 31.3% and 12.7%, respectively [9]. The high stockpiling rate of 91.4% for accompanying dishes among the group companies in this study might reflect their advanced preparedness in terms of BC (Table 3). The percentage of the companies that stocked confectionery (82.9%) was also higher than that reported in the previous study (54.4%) [9]. Although the food stocks shown in this study were more fulfilling than those found in universities [29,30] and a nursery school [31], 81.3% of the participants thought that they should improve their food stocks after they actually ate them in the program.

### 4.3. How the Food Stocks should be Improved?

#### 4.3.1. Disposable Tableware and Cutlery

For the improvement, the most common answer was “stock or increase disposable tableware and cutlery (chopsticks, spoons, and forks)” (48.0%) (Table 5). This would be due to the wide variety of foods stocked (Table 4). For example, as in most local governments [32], if only staple foods such as canned bread or hardtacks are stocked, tableware and cutlery are not necessary because these items can be eaten by hand. In that case, however, nutrient contents and consumer satisfaction might be low. For employees’ health, it is very important to prepare a set of staple foods and accompanying dishes, as observed in this study [33]. Since the necessity for tableware and cutlery will not be noticed unless individuals actually try to eat the stocked food, our training program could make participants think about the necessary preparations.

#### 4.3.2. Side Dishes and Luxury Foods

The second most chosen response was “increase the variety of side dishes” (44.0%) (Table 5). Open-ended responses revealed that they wanted “soup and salad,” “vegetables,” and “less salty foods.” In a previous study that had students live on only foods stockpiled by the university for a two-day period, students also complained that the stockpiled foods were salty and hardly contained any vegetables [34].

For snacks, it can be said that coffee and confectioneries that are consumed daily are preferred to “sticky rice with red bean filling,” “fried *mochi* covered in soy sauce and wrapped in seaweed,” and “bean jelly” (Table 4). They are traditional Japanese foods, but we have few opportunities to eat them in our daily lives. A volunteer who cooked for the victims by the Kumamoto Earthquake reported, “The coffee and sweets that I served between meals were unexpectedly popular” [35]. Habitually consumed familiar foods are most appreciated and suitable in disasters [36].

#### 4.3.3. Cooking Utensils and Heat Sources

The third most frequent answer for the improvement of stockpiles was “stockpile or increase the cooking utensils (pots or kettles)” (40.0%) (Table 5). In this study, the percentage of companies that stocked heat sources was 78.8%, which was higher than the 48.8% found in the previous study [9]. In the previous study, however, no questions were asked about the stockpiling of cooking utensils. Although the stockpiling of heat sources such as cassette stoves is recommended in the national and municipal disaster prevention guidebooks [37,38], there is no description on cooking utensils, and therefore, their stockpiled rate is expected to be low. In households, cooking utensils are generally present for daily use, but in workplaces, the situation is different. In recent years, electric pots and water servers have often been used there and the number of office buildings with a staff kitchen has reduced, resulting in no possession of cooking utensils such as kettles. Indeed, a college student who attended a symposium on stocks for BC commented, “Gas, pots, and water are necessary when boiling water with a cassette stove, but I never come across the availability of pots” [2]. Therefore, it can be presumed that the level of recognition for stockpiling cooking utensils is low. In humanitarian response, there is an indicator that group of four to five individuals has access to two family sized cooking pots [39]. This time, however, only two pots were provided for 33 participants. Since only simple cooking, like boiling water, was required, no confusion was observed (Table 10).

### 4.4. What is Important when Selecting Food Reserves?

The fact that “able to eat hot meals” and “tastiness” were the top two responses was consistent with a previous study that used the same options [22]. Although the training was carried out in midsummer, “able to eat hot meals” was chosen the most. In the majority of the buildings in the central business districts in Japan, however, the maximum operating time on the emergency power supply is 24 h or less [40]. Moreover, the emergency power will be supplied to more important business tasks rather than microwave ovens or electric pots for heating foods and boiling water. Therefore, cassette stoves and cylinders and disaster foods with exothermic agent should be stocked for hot meals.

“Tastiness” of stocked foods is important for consumers, but it is often ignored by buyers whose main concern is cost. Our study also showed that “budget” was the second biggest barrier to improvement in food stockpiles. It is reported that consumers’ needs and logistical convenience is often compete in emergency response [41].

The third most frequently chosen item, “easy clean up” (59.4%), was the 12th chosen answer (20.0%) in the previous study [22]. In the previous study, each female university student received the university’s food stocks and consumed them alone at their desk in the laboratory or at their home. However, in a congregate meal setting, such as that used in this program, a problem with cleaning up could arise. In this study, trash bags were set up inside the training room and filled ones were collected in the corner. Although the meal training consisted of only four meals, from the comments of hygiene team (Table 10), we found that the workload in waste management was large. In this meal training, no-one cooked from scratch. Therefore, the amount of raw waste was smaller than usual cooking and raw waste was mainly derived from leftovers. Since one team member was worried about the odor and insects, serving size of disaster foods should be fun size to prevent leftovers.

Although “storage space” was the biggest barrier to improvement in food stockpiles, a participant pointed out that it will be a problem even after eating, that is, wastes from leftovers, containers, and packages of disaster foods (Table 10). Manufactures are required to develop compact and compostable packages to reduce the plastic wastes. It has been reported that even evacuation centers do not have garbage collection [42]. Waste problems in business establishments during disasters need attention for BC.

### 4.5. Overnight Training and Stockpiles of Bedding

Whereas the stock rate for foods was 91.4%, that for bedding was only 62.5%. There were several people who complained of sleeplessness and poor physical conditions the next morning, indicating that it was hard to stay even one night with currently stocked bedding. The overnight training served as a valuable opportunity to think about the stockpiling of bedding whose current level of preparation was evaluated lower than meals and toilets. According to the Sphere Handbook, the minimum standard for bedding in a humanitarian response is that all affected people have a combination of a blanket and mat [39]. In the participants’ companies, however, the stock rate of mats was as low as 30.0%. Since targeting the well-being of employees is a low-cost strategy that can ensure positive functioning throughout a lengthy recovery period [43], investment in the stockpile of bedding is worthy.

According to Table 9, those who participated in the overnight training came to realize the importance of mats, so the proportion of those who replied that “mats” should be stocked as bedding in the future was significantly higher than that of those who did not stay. On the other hand, “cardboard bed” was chosen more by the participants who did not stay overnight. It has been reported that introduction of cardboard beds in shelters significantly reduced secondary health damages [44,45]. In addition, cardboard beds might be preferable because they provide divided personal space for each person, and their importance could be easily imagined even in those without any experience of using them or being affected by a disaster. For the participants in overnight training, however, the hardness of the floor was a more crucial problem than having no privacy.

### 4.6. Water

The most severe problem for people affected by earthquakes is access to water for drinking/cooking and toilet flushing [46]. An adequate amount of safe water is necessary to prevent death from dehydration, to reduce the risk of water-related disease, and to provide for consumption, cooking, and personal hygienic requirements [47]. The median amount of water stockpiled was nine liters, which is consistent with the guideline of three liters per day per person [37]. In contrast, the amount remaining from the provided three liters of water was 2274 ± 472 mL. The amount of water used was proportional to the time that the participants spent in training, that is, with or without an overnight stay. It is possible that they also drank beverages from PET bottles which they brought along separately from the three liters of water that were distributed, resulting in the low amount of water consumed. In addition, there were many people who used wet tissues instead of water to clean their hands (Table 7), indicating the convenience and necessity of stocking up on wet tissues. The importance of wet tissues was also reported by nurses working in the area affected by the Great East Japan Earthquake [31].

On the other hand, to prevent diarrheal disease which is common in disasters due to limited access to water, handwashing with soap is recommended [48]. The cause of the high prevalence of deep vein thrombosis (DVT) in disaster shelters was supposed to be dehydration due to vomiting and diarrhea experienced by the evacuees because of a shortage of clean water to wash their hands [49]. Although enough wet tissues were available, some participants wanted to wash their hands with water and were concerned about sanitation (Table 7). The Sphere Project recommends providing an adequate supply of water for hand washing and hygienic seal mechanisms [39].

### 4.7. Disaster Toilets

We found that use of disaster toilets due to the ban of flushed toilets caused two problems. One was its contribution to the large quantity of waste (Table 10). Another was reduced desire to take water and food in order to refrain from using disaster toilets (Table 7). It is well known that dehydration caused by reduced water and food intakes is a significant risk factor of DVT [49]. To prevent DVT, it is important to provide enough clean and comfortable toilets. Although the stock rate of disaster toilets was 90.0%, we did not ask the amount. The World Health Organization standard for toilet volume is one latrine for 20 people [50].

### 4.8. Limitations

The biggest flaw of this study was small sample size. This is because we targeted executives who are too busy to participate in the training and their older age could make them hesitate to stay overnight because of their physical conditions. However, since leadership and culture in adopting and promoting preparedness strategies are predominant themes, executives are the primary target for disaster training [51]. By including the stakeholders in the training, support for the preparedness can be obtained incrementally [52] (Green-Baase, 2008). In addition, the small sample size made it possible for us to analyze qualitative data thoroughly. For example, the importance of the initial move could be extracted from participants’ comments shown in Table 10.

Since they were executives and they usually worked as leaders, everyone moved autonomously without the existence of a leader or instruction. When we target other classes, we should modify the contents of the training. For example, although we allocated work to each team this time, without the designation of teams could raise leadership by encouraging participants to learn on their own and think for themselves.

## 5. Conclusions

Testing has always been a component of BCP. This study tested if the current resources were met by the participants’ needs through a disaster training. Seventy-five percent of the participants’ companies had food stocks for three-day as instructed by the Tokyo Metropolitan Government but 81% of the participants thought it is better to be improved after eating stocked meals four times in a row. The stock rate for bedding was 63% but less than 30% of them stocked both blankets and mats as guided by the Sphere Standards. There were several people who complained of sleeplessness and a poor physical condition the next morning and this could be an obstacle in BC. Items and quantity of stockpiles should be determined after the BC personnel used them in similar condition with a disaster. Although the business sectors have had a key role in recent disaster response and recovery, and their resources, both humans and supplies, can be used to provide logistics assistance and coordination for post-disaster needs [53], present preparedness was not enough for the BC personnel to make a significant contribution to their companies and the society. PDCA (plan–do–check–act) cycle should be established for management of stockpiles. Periodical researches that test the appropriateness of the current stockpiles should be repeated for the next renewal of them.

## Figures and Tables

**Table 1:** Contents of the training program.

Time	Contents	Illumination	Flush Toilet	Water Supply in Wash Basin
**Day 1: Thursday, 13 September 2018**				
**9:30**	Reception starts			
**10:00–11:00**	Lecture on disaster food	Available	Available	Available
**11:00–11:15**	Break			
**11:15–12:00**	Orientation, setup	NOT available	NOT available	NOT available
	Meal training (lunch), toilet training			
**12:00–17:30**	Lecture on business continuity with break	Available		
**17:30–18:30**	Meal training (dinner), toilet training	NOT available		
**18:30–19:30**	Overnight training (layout/setup)		Available	Available
	Return home (reflection sheet to be submitted by those who were not staying overnight)			
**19:30–22:00**	DVD viewing (*Shin Godzilla*)			
**22:00**	Lights off			
**Day 2: Friday, 14 September 2018**				
**7:00**	Wake up		Available	Available
**7:00–9:30**	• Clean up the bedding, return the desks to the island layout	NOT available		
	• Meal training (breakfast)			
	(Reflection sheet to be completed and submitted by those who stayed overnight)			
**9:30**	Reception starts (for participants on the 2nd day)			
**9:30–10:00**	Break (those who stayed overnight)			
**10:00–12:30**	Lecture on business continuity with break	Available		
**12:30–13:30**	Meal training (lunch)	NOT available		
**13:30–15:00**	Lecture on business continuity with break	Available		
**15:00–15:10**	Break	NOT available		
**15:10–17:00**	Reporting of results on the reflection sheet	Available		
	Group work, summary			
**17:00–17:30**	Clean up			
**17:30**	End			

**Table 2:** Work assigned to the teams.

Team (No. of People)	Work Description
Lighting (4)	Load batteries in lanterns and install them where needed.
Men’s toilet (4)	Install disaster toilets and explain how to use them. Dispose of waste bags. Install sanitary goods (alcohol sprays and wet tissues) when washing hands with water is not possible.
Women’s toilet (3)	
Meal (4)	To the participants who did not bring along their companies’ food stocks, give four meals prepared by the secretariat (1st meal: dried bread; 2nd meal: pregelatinized rice; 3rd meal: pregelatinized rice; 4th meal: rescue foods). Install cassette stoves/cookware and clean up the cooking tables.
Hygiene (4)	Install trash bags in the classroom and sort trash (including leftovers). Keep it neat and tidy and maintain hygiene standards.
Accommodation (5)	Check the number of people who are staying overnight. Check the inventory and distribute bedding. Lay bedding out for the overnight stay. Give instructions to clean up the bedding the next morning.
Public relations (5)	Assess needs and request necessary items. Negotiate with the secretariat.
Other (4)	Identify whether there is any other work required and act accordingly, including helping other teams.

**Table 3:** Food items stockpiled at the participants’ companies (*N* = 35).

(Multiple Answers Allowed)	*n*	%
1. Staple foods (dried bread, pregelatinized rice, canned bread, porridge)	32	91.4
2. Accompanying dishes (canned meat and fish, soup)	32	91.4
3. Drinks other than water (canned juice)	20	57.1
4. Confectionery (Calorie Mate, bean jelly, cookies)	29	82.9
5. Others	2	5.7
6. There is no food stock	1	2.9

**Table 4:** Frequently observed combination of stocked foods that participants brought along.

September 13 Lunch	September 13 Dinner	September 14 Breakfast	September 14 Lunch	Snacks	No. of Observations ^1^
Mushroom rice	Seaweed rice	Long-life bread	Brown seaweed rice	Sticky rice with red bean filling	5 participants
Sardines boiled in soy sauce	Hamburger steak	Vegetable juice	Chicken stew		
Curry and rice	Rice cooked with various ingredients	Long-life bread	Mushroom rice	Fried mochi covered in soy sauce and wrapped in seaweed	4 participants
	Mackerel simmered in miso sauce	Vegetable juice	Sardines boiled in soy sauce		
Mushroom rice	Seaweed rice	Long-life bread	Brown seaweed rice	Sticky rice with red bean filling	2 participants
Sardines boiled in soy sauce	Hamburger steak	Vegetable juice	Ginger-grilled saury		
Brown seaweed rice	Rice cooked with various ingredients	Long-life bread	Curry and rice	Boiled rice cake covered with sweetened yellow soybean flour	2 participants
Saury simmered in ginger	Meat and potato stew	Vegetable juice			
Curry and rice	Rice cooked with various ingredients	Long-life bread	Mushroom rice	Fried mochi covered in soy sauce and wrapped in seaweed	2 participants
	Mackerel simmered in miso sauce	Vegetable juice	Sardines boiled in soy sauce		
Brown seaweed rice	Rice cooked with various ingredients	Long-life bread	Curry and rice	Boiled rice cake covered with sweetened yellow soybean flour	2 participants
Saury simmered in ginger	Meat and potato stew	Vegetable juice			
Pregelatinized rice	Rescue foods	Canned bread	Pregelatinized rice	Bean jelly	2 participants
				Biscuit bars	
Disaster food	Disaster food	Udon noodles	Disaster food	Biscuits	2 participants

^1^ Only combinations that two or more people brought are shown.

**Table 5:** How the current food stocks should be changed (*N* = 25).

(Multiple Answers Allowed)	*n*	%
Stock staple foods (or increase the quantity)	2	8.0
Increase the variety of staple foods	9	36.0
Stock accompanying dishes (or increase the quantity)	8	32.0
Increase the variety of side dishes	11	44.0
Stock water (or increase the quantity)	8	32.0
Stock heat sources (or increase the quantity)	9	36.0
Stock cooking utensils (pots, kettles) (or increase the quantity)	10	40.0
Stock disposable tableware and dishes (chopsticks, spoons, forks) (or increase the quantity)	12	48.0
Open-ended responses (selected)		
✔ It would be even better if something like soup or salad were available.		
✔ It would be better to stock powdered coffee and tea bags. I get tired of beverages that consist of water only.		
✔ It would be even better to add luxury food and drink like sweets and coffee.		
✔ Are there enough vegetables (vitamins, fibers)?		
✔ Too salty.		
✔ I think that would be better to increase white rice and those foods that contain less salt.		

**Table 6:** Important points in the selection of stockpiled foods (*N* = 32).

(Multiple Answers Allowed)	*n*	%
Able to eat hot meals	25	78.1
Tastiness	24	75.0
Easy cleanup	19	59.4
No tableware required	17	53.1
Hygienic	17	53.1
High nutritional value	13	40.6
Variety	10	31.3
Vegetables are contained	10	31.3
Compact storage	9	28.1
Low price	8	25.0
Long shelf life	7	21.9
Meat and fish are contained	6	18.8
Large serving size	2	6.3
Small serving size	2	6.3

**Table 7:** Impressions and observations of living without using running water and electricity.

*Water (12)*
I realized the importance of water (necessary for hand washing, heating retort-pouched food)
I realized the inconvenience when I could not wash my hands and when the toilet could not be flushed. I could endure it because it was only for a short time on this occasion, but I would have been concerned about bacteria and other sanitation issues if it had extended over a longer period of time.
*Wet tissues (7)*
I realized the importance of wet tissues when my hands were stained by food.
I think that it is important to stock up on wet tissues.
*Toilet (3)*
I felt that the toilet was laborious and troublesome.
If the toilet could not be used, the desire to take water and food would decrease.
*Lighting (17)*
The small letters were hard to see (the instructions on how to use the toilet were in small print, and it was dim inside the compartment).
It was necessary to live together as close as possible with a small lantern.
*It would be tougher if the period got longer (3)*
I have never experienced living without using water or lights. It would be all right for just one night and day, but I think it would be tough for three or four days.
Although I could tolerate it for a short time, it would get mentally harder if the period got longer, and work would likely be impeded.
*No trouble (3)*
I participated in boy scout activities as a child, so the environment did not feel uncomfortable.
I did not really feel the inconvenience because the time was short.
*Valuable experience (2)*
I once again felt the gratitude of being supplied with these in our normal lives. I also realized the importance of preparing in advance.

*N* = 27. Figures in parenthesis show the number of similar quotes. One cell shows the response from one person. To showcase the concrete content of each category, one to two quotes are shown as examples.

**Table 8:** Stockpiling of bedding at participants’ companies.

Preliminary Questionnaire							Reflection Sheet		
Current Status on the Stockpiling of Bedding (*n* = 30)	*n*	%		Currently Stockpiled Items (*n* = 20)	*n*	%	What Should Be Stockpiled in the Future (*n* = 21) ^1^	*n*	%
Stockpiled	20	62.5	⇒ ^2^	Blankets	11	55.0	Blankets	14	66.7
Not stockpiled	2	6.3		Mats	6	30.0	Mats	13	61.9
I do not know	10	31.3		Aluminum blankets	6	30.0	Cardboard beds	10	47.6
				Sleeping bag sheets	4	20.0	Sleeping bag sheets	8	38.1
				Cardboard beds	1	5.0	Pillows	7	33.3
				Pillows	0	0.0	Aluminum blankets	4	19.0

^1^ Responses were received from 21 of the 22 participants, who responded, “Bedding should be stockpiled or the stocked items should be changed in the future.” ^2^ Responses to “currently stockpiled items” were received from 20 participants who responded, “stockpiled” to “current status on the stockpiling of bedding.”.

**Table 9:** Thoughts and observations after having stayed overnight.

*Layout, environment (5)*
Although consideration was given to privacy, such as using the desks as partitions, I do not believe the accommodation environment would be adequate if we assume that disaster recovery work is going to be carried out the next day.
*Noise, snoring (5)*
I could not sleep as much as I wanted to. I was bothered by the surrounding sounds.
*Difficulty in sleeping (4)*
I think I might have been able to get to sleep if I had gotten used to it, but I felt that it was going to be tough for the first 2–3 days.
*Mats (4)*
The mat did not inflate to 2.5 cm as written in the user’s manual, and my physical condition became worse in the morning because of the flimsy mat.
*Room temperature (4)*
The air temperature was not a problem, particularly as the training was conducted in September, but I am concerned about winter as I do not know what would happen in December, January, and February.
*Body pain (3)*
My body ached, and I woke up midway through the night.
*Sleeping bag sheet (2)*
I got to know what a sleeping bag sheet is for the first time, but it was comfortable.
*BC (2)*
I could not get enough rest even in a relatively good environment, and I felt uneasy about taking on long-term challenges while conducting business continuity activities.

*N* = 17. Figures in parenthesis show the number of similar quotes. Each cell shows the answer from one person. To showcase the concrete content of each category, one quote is shown as an example.

**Table 10:** Thoughts and observations after having carried out the work assigned to the teams.

Team	Free Description
Lighting	Some parts required double the effort if preparations were not made first.
	I tried to illuminate the notes in the bathroom, but the letters were small and could not be seen!
	There will be no commotion as long as the procedure is determined, even if it is just a rough one.
	Reserves were distributed by people who were not in charge of them (voluntarily).
Men’s toilet	***Large volume of waste (3)***
	Regardless of whether it was urine or stool, the waste felt surprisingly heavy.
	As it has unexpectedly large volume, air should be prevented from entering as much as possible when you tie the bag closed after use.
	Evolution of technology (solidifying quickly). Odor is becoming less of a problem due to the performance of the sealed chuck.
Women’s toilet	The initial move is important (grasping and distributing the whole quantity).
	I thought that it would be nice if the instructions on how to use the toilet were pasted in large text (those who are not prepared will find it hard to understand).
	The toilet (in which an absorbent sheet is set in a plastic bag) was very easy to set up.
	Today, I did not feel there was any problem in particular because the number of women was small, but when the number of people is large, I thought that the amounts of waste and odor might be issues.
Meal	***Use of stoves (4)***
	I think that there is a need for a procedure to ensure cooking is carried out efficiently when the number of stoves is small. Fortunately, there was no confusion because not many people used hot water this time.
Hygiene	***Waste sorting (2)***
	Places to take out the trash and how to sort out the trash must be determined and checked (to see if they are full) each time. If the sorting method is not stated, the person throwing away the trash will be confused.
	Instead of raw waste, a lot of plastic bags and other incombustible trash were thrown away.
	For outdoor hygiene, measures to deal with the odor and insects are also necessary.
	The amount of trash is large, so a storage place needs to be secured.
Accommodation	***Sleeping arrangements (2)***
	Since enough stockpiles were prepared in advance for the number of people participating, no excess or deficiency in stockpiles occurred. When determining the sleeping positions, I learned that we must consider various issues, such as securing flow lines and privacy, the efforts required to move desks and chairs, and so on.
	***Things went well (2)***
	Even without the existence of a leader, everyone knew their roles and what they should do, so we managed to set up the accommodation arrangements without any problems. Although we were using the bedding for the first time, everyone was able to use it correctly despite no explanation being given.
	Although the overnight training was conducted on the assumption that it is only for one night, the techniques for tidying up would have to be reviewed for an extended stay.
Public relations	***No work this time (2)***
	There isn’t much use within the scope of this training. In the event of a disaster, I believe information gathering, coordination, and cooperation with local governments and the surroundings will be necessary.
	I understood the importance of allocating the work among the different teams.
Other	***Helped other teams (2)***
	I helped the lighting team and meal team. I felt that it was inefficient unless each team followed the instructions after they decided on a leader first. During the actual exercise, a team leader must be appointed first for the activity.
	I felt that the effort needed to prepare the meals was surprisingly easy.
	The sanitation measures, including the disposal of excretion, were terrible.

*N* = 29. Figures in parenthesis show the number of similar quotes. To showcase the concrete content of the grouping, one quote is shown as an example. One cell shows the answer from one person.

## Appendix A. Preliminary Questionnaire

(Please return the completed survey to the BC Research Centre by 4pm on 6th September)

Office Name		Name	
Telephone		Email	

The third seminar is intended to simulate the actual experience of being in a disaster, and to deepen understanding of the importance of business continuity preparations for companies in a disaster.

Please answer the questionnaire below.

**Q1. Will you be staying this time?**

1. Yes. If you choose this option, next, please go to question 4.
2. No. If you choose this option, next, please answer questions 2 and 3.

**Q.2 For those who answered “no” to Q1: please circle your reason for your answer below (please circle all that apply).**

1. Worried about physical health
2. Do not wish to stay
3. Other: (     )

**Q.3 For those who answered “no” to Q1: what kind of changes would be necessary (if any) for you to want to stay?**

(e.g., if cardboard beds were provided for communal sleeping, or if you were able to eat food that is closer to your usual diet)

**Please answer the following questions regardless of whether you will be staying or not.**

**Q4. Please circle the items that you have stockpiled at your office. (Please circle all that apply).**

1. Food staples (dried bread, cooked rice, tinned bread, porridge etc.)

2. Main dishes (canned meat or fish, soup etc.)

3. Beverages other than water (canned juice etc.)

4. Sweets (Calorie Mate, red bean paste, cookies etc.)

5. Other (     )

6. No food stockpiles available

Note: For items such as Rescue Foods that come in a set of a staple and a main dish, please circle both 1 and 2

**Q.5 How many meals for one person do you have stockpiled? Please enter the number in the parenthesis below.**

                           ( ) meals

Note: For the training on 13th–14th September, please bring 4 meals from the current food stockpile at your office. You will eat what you have brought with you for lunch and dinner on the first day and breakfast and lunch on the second day. If you have no stockpiles or do not have enough stock, disaster food will be prepared for you.

**Q.6 How many individual liters of drinking water do you have stockpiled? Please enter the number in the parenthesis below.**

                           ( ) liters

Note: We will supply water 3 L of water per person per day (total 6 L) to use during the training (for drinking, cooking and day-to-day uses, such as washing) so there is no need to bring your own water.

**Q.7 Do you have a stockpile of heat sources for use in cooking, such as camping stoves?**

1. Yes
2. No

**Q.8 Please provide details of the stockpiled food and cooking method that your office is planning to provide:**

*Example entry*	13th Sept Lunch	13th Sept Dinner	14th Sept Breakfast	14th Sept Lunch	Snacks
Food type	*Cooked rice*	*Cooked rice*	*Dry bread*	*Cooked rice*	*None*
Preparation method	Rehydration/Boiling	Rehydration/Boiling	Rehydration/Boiling	Rehydration/Boiling	Rehydration/Boiling

Note: Water cannot be boiled without providing heat sources.

	13th Sept Lunch	13th Sept Dinner	14th Sept Breakfast	14th Sept Lunch	Snacks
Food type					
Preparation method	Rehydration/Boiling	Rehydration/Boiling	Rehydration/Boiling	Rehydration/Boiling	Rehydration/Boiling

**Q.9 Do you have a stockpile of toilets for use in a disaster?**

1. Yes
2. No

**Table A1:** Stockpile list used in the training.

Classification	Item	Quantity
Illumination	Lantern	16 pieces
	Battery (D size)	48 units
Food and beverage	PET bottle water—3 L per person	2 L × 1 units
		500 mL × 2 units
	Cassette stove	3 units
	Cassette cylinder	3 units
	Pot for boiling	2 units
	Kettle	2 units
	Measuring cup	2 units
	Disposable chopsticks	200 units
	Paper plates	200 sheets × 2 types
	Plastic wrap	2 rolls
Hygiene	Box tissues	3 boxes
	Wet tissues	8 packages
	Paper towels	8 boxes
	Alcohol spray	6 bottles
	Trash bags	3 units
	Bucket	2 units
	Disposable toilet	210 times use
Accommodation	Sleeping bag sheet	33 bags
	Cardboard bed	2 units
	Automatic expansion mat	33 sheets
	Blue sheet	10 sheets
	Blanket	33 sheets
Stationery	Felt pen	1 unit per person
	Curing tape	1 piece
	Scissors	1 piece
	Scotch tape	1 piece
